# Seed DNA damage responses promote germination and growth in *Arabidopsis thaliana*

**DOI:** 10.1073/pnas.2202172119

**Published:** 2022-07-18

**Authors:** Wanda M. Waterworth, Rosalind Latham, Dapeng Wang, Mona Alsharif, Christopher E. West

**Affiliations:** ^a^Faculty of Biological Sciences, University of Leeds, Leeds, LS2 9JT, United Kingdom;; ^b^Leeds Omics, University of Leeds, Leeds, LS2 9JT, United Kingdom;; ^c^Wellcome Centre for Human Genetics, University of Oxford, Oxford, OX3 7BN, United Kingdom;; ^d^National Heart and Lung Institute, Imperial College London, London, SW3 6LY, United Kingdom

**Keywords:** seed, germination, DNA repair, genome stability, seed quality

## Abstract

The desiccated, quiescent state of seeds confers extended survival of the embryonic plant. However, accumulation of striking levels of genome damage in quiescence impairs germination and threatens plant survival. The mechanisms by which seeds mitigate this damage remain unclear. Here, we reveal that imbibed *Arabidopsis* seeds display high resistance to DNA damage, which is lost as seeds advance to germination, coincident with increasing cell cycle activity. In contrast to seedlings, we show that seeds minimize the impact of DNA damage by reducing meristem disruption and delaying SOG1-dependent programmed cell death. This promotes root growth early postgermination. In response to naturally accumulated DNA damage in aging seeds, SOG1 activates cell death postgermination. SOG1 activities are also important for promoting successful seedling establishment. These distinct cellular responses of seeds and seedlings are reflected by different DNA damage transcriptional profiles. Comparative analysis of DNA repair mutants identifies roles of the major genome maintenance pathways in germination but that the repair of cytotoxic chromosomal breaks is the most important for seed longevity. Collectively, these results indicate that high levels of DNA damage incurred in seeds are countered by low cell cycle activity, cell cycle checkpoints, and DNA repair, promoting successful seedling establishment. Our findings reveal insight into both the physiological significance of plant DNA damage responses and the mechanisms which maintain seed longevity, important for survival of plant populations in the natural environment and sustainable crop production under changing climates.

## Significance

Successful germination underpins crop production and natural ecosystems. However, the desiccation-tolerant seed accumulates striking levels of genome damage in quiescence associated with seed aging. Here, we show that seeds display intrinsic resistance to genome stress, which is lost as seeds advance to germination. Seeds minimize meristem disruption and delay programmed cell death in response to seed aging to promote root growth early postgermination. This reveals distinct responses of seeds to DNA damage in terms of growth and transcriptional profiles, which support rapid seedling establishment at this crucial stage of the plant lifecycle. These findings advance our understanding of both plant DNA damage responses and seed longevity, important for crop yields and plant survival under changing climates.

Genome maintenance is critical for plant growth and safeguards transmission of genetic information. DNA repair and response mechanisms function to mitigate the mutational and growth-inhibitory effects of DNA damage induced by a wide range of environmental and endogenous cellular factors (1). In particular, DNA double-strand breaks (DSBs) represent highly cytotoxic forms of DNA damage, potentially resulting in chromosome fragmentation, loss of genetic information, and cell death if unrepaired. In eukaryotes, sensing of severe forms of DNA damage results in the activation of cellular DNA damage responses (DDR) that maintain genome stability. The DDR in plants is orchestrated by the protein kinases ATAXIA TELANGIECTASIA MUTATED (ATM) and ATM AND RAD3-RELATED (ATR), which activate downstream responses, including DNA repair and cell cycle checkpoints, to maintain genome integrity and minimize formation of mutations (2, 3). In plants, high levels of damage induce programmed cell death (PCD) in meristems, which represent the progenitors of plant organs (4–6). Signaling from both ATM and ATR is integrated by the transcription factor SUPPRESSOR OF GAMMA 1 (SOG1), which is unique to plants but functionally similar to the mammalian tumor suppressor p53 (5). In response to genotoxic stress, SOG1-mediated PCD provides an effective mechanism for the selective elimination of cells with compromised genomes in meristems (7).

Previously, we established important roles for genome maintenance in seed germination and longevity (8, 9). Successful germination, a critical developmental transition in the plant lifecycle, is crucial for both plant survival in the natural environment and crop productivity. Desiccation-tolerant (orthodox) seeds, typical of major staple crops, undergo programmed desiccation during the maturation phase of seed development (10). Cellular metabolism is reduced to very low levels, leading to embryo quiescence in the desiccated seed. This anhydrobiotic state functions as an effective mechanism to prolong seed viability. However, seed germination potential declines over time and under adverse environmental and storage conditions, impacting germination performance and ultimately culminating in viability loss (11). Seed longevity is determined by the complex interaction of environmental and genetic factors, and remains incompletely understood at the molecular level (11). However, the low activity of cellular maintenance pathways in the desiccated state is associated with deterioration of cellular structures and biological macromolecules, including nucleic acids and proteins (9, 12–14).

Seed germination initiates with imbibition (water uptake) and is completed upon emergence of the young root (radicle) from the seed coat (10). In *Arabidopsis*, germination coincides with increased cell cycle activity, including DNA replication and cell division (15). DNA damage accumulated in the quiescent embryo must be repaired prior to cell division in order to minimize mutagenesis and inhibition of seedling growth and development (8). Activation of cellular DDR pathways and DNA repair activities are initiated early in seed imbibition, preceding initiation of the cell cycle by several hours (9, 16). Radicle emergence is progressively delayed as vigor declines in aged seeds and is accompanied by an extended period of genome repair (16). The ability to withstand or repair genome damage is crucial to seed germination vigor and viability (17), revealed by the aging hypersensitivity of *Arabidopsis* seeds deficient in the factors required for nonhomologous end-joining (NHEJ) of DSBs (9). In addition, repair of base damage by base excision repair (BER) affects seed longevity, with mutants displaying reduced vigor, whereas overexpression lines are resistant to aging (18, 19). Loss of seed vigor and viability is accompanied by elevated frequencies of cytogenetic abnormalities, including chromosome fragmentation and anaphase bridges (20) that result from chromosomal fusions produced by misrepair of DSBs. ATM and ATR regulate germination in response to DNA damage incurred during aging, with aged *atm* seeds germinating more rapidly than wild type but displaying extensive chromosomal abnormalities (8). ATM links activation of the cell cycle with sensing of genome integrity, thereby imposing a lag period to germination as vigor declines (8). This identifies that the DDR is active in imbibing seeds, but it is unknown how plants mitigate the effects of the high levels of genotoxic stress incurred in quiescence on germination and seedling growth.

Here, we reveal that imbibed *Arabidopsis* seeds display striking resistance to the effects of DNA damage relative to seedlings, coincident with low levels of cell cycle activity in seeds, but this resistance is lost as seeds advance to germination. This reveals differences in plant DDRs dependent on developmental stage, which are reflected by distinct transcriptional DDRs in seeds and seedlings. We demonstrate that PCD is mediated by SOG1 in response to naturally incurred DNA damage in aged seeds but PCD occurs with low frequency prior to germination. Comparative genetic analysis establishes that all major DNA repair pathways are determinants of seed longevity, but that repair of DSBs is most important to germination and seed quality, reflecting the high cytotoxicity of these lesions. Collectively, these studies lead to a model whereby the detrimental effects of DNA damage incurred in the quiescent seed are mitigated by the low cell cycle activity and DNA repair pathways operative early postimbibition. We propose that DDRs in seeds facilitate both the preservation of genome integrity and promotion of successful postgerminative growth and seedling establishment. Understanding the mechanistic basis of seed longevity is important for sustainable crop production under climate change and fundamental to the preservation of plant germplasm resources in seedbanks.

## Results

### Imbibed Seeds Display an Intrinsic Resistance to DNA Damage.

**Fig. 1.**
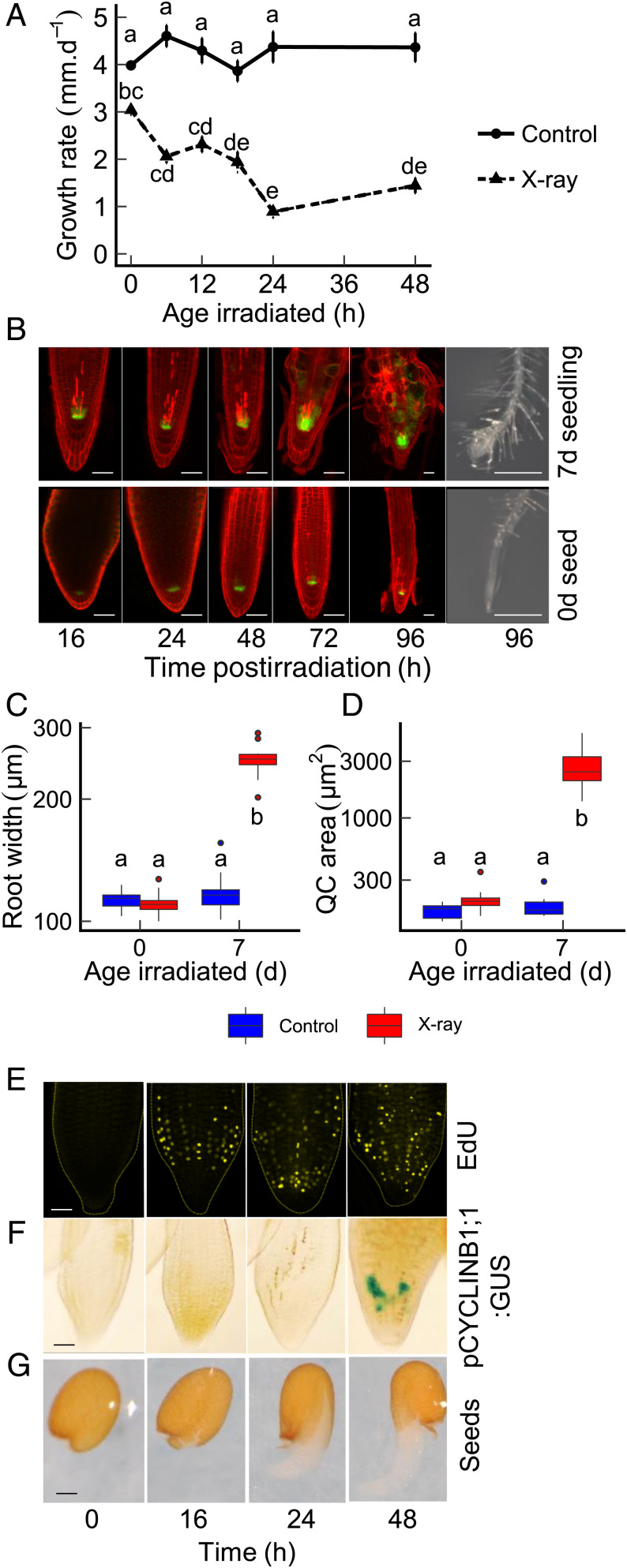
Imbibed seeds progressively lose radioresistance through germination. (*A*) Root growth sensitivity of wild-type Col-0 *Arabidopsis* seeds in response to X-irradiation. Seeds were stratified for 2 d at 4 °C and exposed to X-rays (100 Gy at 2 Gy/min) at the stated time point after transfer to 23 °C. Irradiated seeds and unirradiated controls were plated onto half MS plates postirradiation, grown vertically at 23 °C 16-h day, and root growth quantified over 7 d. Data were analyzed using one‐way ANOVA with Tukey's honestly significant difference post hoc test. Letters denote homogeneous subsets (*P* < 0.05). Error bars show SEM of >15 roots. (*B*) X-ray–induced PCD in *Arabidopsis* Col-0 seeds and seedlings. Seeds were stratified for 2 d at 4 °C and either exposed to 100-Gy X-rays immediately poststratification (0-d seeds) or after 7 d growth on half MS at 23 °C 16-h day (7-d seedlings). The appearance of cell death was monitored over 96-h recovery from irradiation. Confocal images of PI-stained Col-0 roots expressing the PWOX5:GFP QC marker. (Scale bar, 50 µm.) A bright field image is also shown at 96 h. (Scale bar, 500 µm.) See *SI Appendix*, Fig. S3 for unirradiated controls. (*C*) Maximum width of the root tip (*n* = 10) and (*D*) area of WOX5-GFP expression in plants 96 h after exposure to 100-Gy X-rays at 0 d or 7 d poststratification or unirradiated controls (*n* = 10). Letters denote homogeneous subsets (*P* < 0.001). (*E*) Timing of S-phase (DNA replication) in germinating Col-0 *Arabidopsis* seeds. EdU labeling and confocal microscopy of germinating seeds indicating the onset of S-phase by 16 h. (*F*) Timing of G2/M-phase in germinating Col-0 *Arabidopsis* seeds. β-glucuronidase (GUS) reporter analysis of PCYCLINB1;1-GUS lines indicating G2/M-phase cells postgermination at 2 d poststratification. (*G*) Representative brightfield images of seeds at different time points poststratification. (Scale bar, 50 µm.)

Our previous analysis revealed that desiccation-tolerant seeds incur high levels of DNA damage during desiccation and quiescence (8, 21). This led to the hypothesis that desiccation-tolerant seeds can withstand severe genotoxic stress and mitigate the effects of genome damage on subsequent plant growth. To test this hypothesis, seeds were imbibed in dH_2_O and incubated for 2 d at 4 °C to synchronize germination in a process termed stratification. Seeds were then transferred to 23 °C and irradiated with 100-Gy X-rays (2 Gy/min) at different time points (0–48 h) after transfer. Seeds were plated on half Murashige and Skoog Basal Medium (MS) agar, and root growth of the emergent seedling was monitored, relative to unirradiated controls (Fig. 1*A*). Germination was not affected by this dose of irradiation, but subsequent root growth was reduced, dependent on germination stage at which seeds were X-rayed. While unirradiated seeds displayed similar mean seedling root growth rates over the 7-d period, irradiated seeds displayed increasing sensitivity to X-rays as germination progressed, observed as reduced rates of seedling root growth when irradiated at later growth stages (*P* < 0.001, two-way ANOVA). Furthermore, quantification of the fresh mass of above-ground tissue 3 wk postirradiation demonstrated that the resistance to genome damage observed early in germination resulted in long-term enhancement of plant growth; irradiated seedlings displayed a significantly greater reduction in biomass relative to seeds (*SI Appendix*, Fig. S1; *P* < 0.001, two-way ANOVA). These results reveal that seeds display increased resistance to the effects of DNA damage during the early phases postimbibition.

The response of seeds to genotoxic stress was further analyzed by investigating the incidence of cell death in seeds, a hallmark of the plant DDR. In plants, DNA damage-induced PCD, mediated by SOG1, occurs in the stem cell initials of the root apical meristem (RAM) within 8–12 h of exposure to high-energy radiation (5). PCD functions to eliminate cells with compromised genomes from meristems, maintaining meristem integrity (22). Wild-type and *sog1* mutant lines were analyzed for X-ray–induced PCD during germination, visualized by propidium iodide (PI) staining and confocal microscopy. Mutant *sog1-2* and *sog1-3* alleles were generated in the Col-0 genetic background using CRISPR-Cas9 mutagenesis (23) (*SI Appendix*, Fig. S2). Seeds were stratified for 2 d at 4 °C before irradiation for 30 min at room temperature (RT). Cell death was monitored at various time points up to 96 h after the end of the treatment, compared to unirradiated control (Fig. 1*B* and *SI Appendix*, Figs. S3 and S4). While cell death was observed in irradiated 7-d seedling RAMs by the first time point at 16 h, the RAM of irradiated 0-d seeds displayed little incidence of PCD until 72 h postirradiation. *sog1* mutant lines displayed low incidence of PCD at either developmental stage, although after 2 d postirradiation, dead cells were observed distal to the stem cell initials, as previously reported (*SI Appendix*, Figs. S3 and S4) (7). The low incidence of PCD in the seed embryo RAM is indicative of significant differences in the plant DDR between seeds and seedlings. Furthermore, previous studies of irradiated seedlings reported root meristem disruption and expansion of WOX5 expression, a marker of the quiescent center (QC) that consists of slowly dividing stem cells, within 3 d of irradiation (7). These features were also observed in 7-d seedlings at 96 h post–X-ray with significant expansion of the RAM and WOX expression (*P* < 0.001, Fig. 1 *B*–*D*). In contrast, roots from irradiated 0-d seeds displayed no significant increase in meristem width or WOX5 expression during subsequent seedling growth. In seeds, low levels of PCD may result from the reduced cell cycle activity of imbibed embryos early in germination (15). We analyzed cell cycle activity in germinating seeds. Initiation of S-phase (DNA replication) was monitored by incorporation of the thymidine nucleoside analog ethynyl deoxyuridine (EdU). In addition, *PCYCLINB1;1:CYCLIN(1-116)-GUS* activity was used to identify cells in G2 and G2/M (24). We observed that S-phase activity was not detectable in embryos immediately poststratification, but EdU labeling was detectable by 16 h after transfer to 22 °C (Fig. 1*E*). Evidence of G2/M cells was not detected until postgermination at 48 h (Fig. 1*F*). The transcriptional profile of seeds immediately poststratification was compared to 7-d seedlings, confirming significantly lower cell cycle gene expression pregermination (*SI Appendix*, Fig. S5, *P* < 0.001 and Dataset S1) in line with previous reports (15). Taken together, these results are consistent with low cell cycle activity in imbibed seeds resulting in low levels of PCD and protection from the growth-inhibitory effects of DNA damage. This indicates that plants have distinct physiological responses to DNA damage, dependent on developmental stage and reflecting the underlying cell cycle activity.

### The Transcriptional DDR Is Dependent on Plant Developmental Stage.

**Fig. 2.**
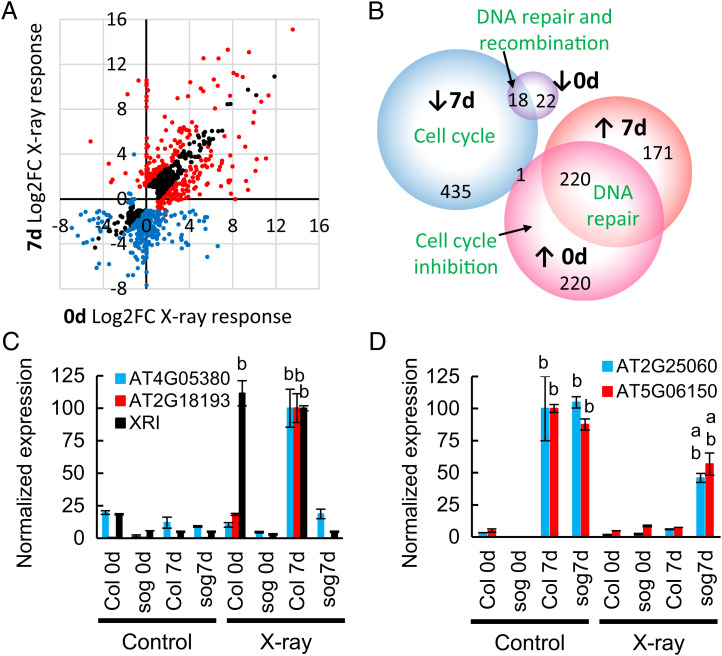
The *Arabidopsis* transcriptional response to DNA damage differs between developmental stages. RNAseq analysis of *Arabidopsis* Col-0 seed and seedling responses to X-rays. Seeds (0 d) were stratified on half MS agar for 2 d at 4 °C before X-irradiation (100 Gy at 2 Gy/min at RT). Seedlings (7 d) were grown vertically on half MS plates at 23 °C 16-h day for 7 d before X-irradiation as described for seeds. Unirradiated controls were maintained at RT for 30 min in place of X-ray treatment. RNA was extracted from stratified seeds or seedlings 6 h after the end of the irradiation treatment, and transcripts were quantified by sequencing. (*A*) Comparison of the log2 fold change (FC) in transcript levels of genes differentially expressed 6 h post 100-Gy X-irradiation in 7-d seedlings and stratified seeds (0 d) by RNAseq. X-ray–responsive genes with no significant difference in transcript levels between 0-d and 7-d irradiated samples (black). Genes with lower (<−1 log2 fold change) (blue) and higher (>1 log2 fold change) (red) abundance postirradiation and which are differentially expressed (*P* < 0.05 Holm-Bonferroni correction) between irradiated 0-d and 7-d developmental stages are indicated. (*B*) Venn diagram displaying the irradiation response in 0-d seeds or 7-d seedlings. Genes that are induced by irradiation are indicated (↑) in seeds (pink circle, 0 d) or seedlings (red circle, 7 d) or at both stages. Genes with lower (↓) abundance after irradiation in seeds (0 d) are indicated by the purple circle and in seedlings (7 d) by the blue circle. The gene ontology enrichment of highest statistical significance is indicated in green text, and numbers of genes in each section of the Venn diagram are indicated. (*C*) qPCR confirmation of RNAseq expression patterns for transcripts with higher abundance after irradiation, including *XRI1*, induced in 0-d seeds and 7-d seedlings in WT but not in *sog1-2* mutants, compared to *AT4G05380* and *AT2G18193* that display significant induction only in 7-d WT plants. (*D*) qPCR analysis *AT2G25060* and *AT5G06150* that display significant X-ray–induced reduction in transcript levels only in 7-d WT seedlings. Data were analyzed by ANOVA with Tukey’s post hoc correction for multiple testing (*P* < 0.01) for each gene. All unlabeled data points belong to group “a.”

The low levels of PCD and meristem disruption observed in irradiated seeds indicate intrinsic differences in the DDR between seeds and seedlings, likely resulting from the low cell cycle activity in seeds. The *Arabidopsis* transcriptional DDR is well characterized in seedlings and mature plants from 4- to 33-d growth ((25) and citations therein). Here, we compare the transcriptional DDR 6 h after a 30-min 100-Gy X-ray dose (at RT) between stratified seeds (0 d) and 7-d seedlings (Dataset S2). Of the 1088 genes that displayed significant X-ray responses at either developmental stage (*P* adj < 0.05, >2-fold change), 66% differed more than 2-fold in the response to irradiation in 0-d seeds relative to 7-d seedlings, with only one-third of X-ray–responsive genes displaying similar fold changes in expression at both developmental stages (Fig. 2*A*). Gene ontology analysis identified gene function enrichment in the development stage-specific DDR (Fig. 2*B* and *SI Appendix*, Tables S1–S4 and Fig. S6, *P* < 0.05 Holm-Bonferroni correction). After X-irradiation, both seeds and seedlings displayed significantly increased expression of DNA repair factors (e.g., *RAD51*) and reduced expression of DNA replication and recombination factors (e.g., *BRCA2*). However, the seedling-specific DDR was characterized by a large reduction in cell cycle factors (e.g., *CYCLINB1;4*), not observed in seeds. Genes with increased transcript levels in the seed-specific DDR were significantly enriched in negative regulators of the cell cycle (e.g., *WEE1*). *WEE1* displayed an ∼9-fold induction in X-ray–treated seeds, in contrast to 7-d seedlings, where *WEE1* expression was not significantly induced (1.2-fold, *P* > 0.05) 6 h postirradiation. This is in line with published reports showing little change in *WEE1* transcripts 6 h posttreatment, although an ∼3-fold induction was previously observed 1.5 h postirradiation (25). DNA damage-induced changes in gene expression were confirmed by qPCR for analysis of representative genes displaying specificity of the DDR in seeds or seedlings (Fig. 2 *C* and *D*). Expression of the DDR-associated gene *XRAY INDUCIBLE 1* (26) was shown to be induced by X-rays at both stages of plant development in wild-type but not *sog1* mutants, with similar levels of induction in seeds and seedlings. In contrast, expression of genes *AT4G05380* and *AT2G18193* (P-loop nucleoside triphosphate hydrolases) only displayed significant induction upon X-irradiation in wild-type seedlings and not in imbibed seeds or *sog1* mutants (Fig. 2*C*). In contrast, *AT2G25060* (*EARLY NODULIN-LIKE PROTEIN 14*) and *AT5G06150* (*CYCB1;2*) displayed low basal expression in seeds and failed to display the reduced expression observed in irradiated wild-type seedlings (Fig. 2*D*). Taken with the low PCD and low meristem disruption observed in irradiated seeds, analysis of the DDR reveals that seeds display different transcriptional responses to DNA damage compared to seedlings, underlying their distinct physiological responses to genome stress.

### SOG1 Regulates Germination in Response to Aging.

**Fig. 3.**
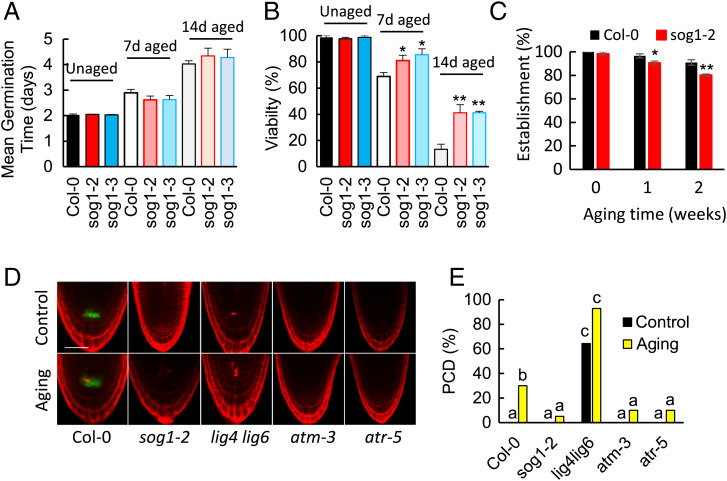
Germination and aging-induced cell death in Col-0 and *sog1* mutant lines. Germination of *sog1* mutant seed is resistant to aging. Germination of *sog1-2*, *sog1-3*, and Col-0 seeds was analyzed after accelerated aging at 35 °C and 83% RH relative to unaged control seed. Seeds were stratified at 4 °C for 2 d before transfer to 23 °C 16-h day and scored for radicle emergence each day poststratification. (*A*) Mean germination time (MGT) and (*B*) viability. Error bars represent the SEM of the mean of three replicates of 30 seeds (*P < 0.05; **P < 0.01, *t* test of the *sog1* mutant against Col-0 for each aging regime). (*C*) Seedling establishment is lower in seedlings germinated from aged *sog1* mutants relative to wild type. Analysis of survival of seeds transferred from germination to soil at 7 d postgermination. Error bars are SEM of three replicates of 20 plants (*P < 0.05; **P < 0.01, *t* test). (*D*) PCD in wild-type and DNA repair mutant seed was analyzed by viability staining. Embryos were isolated from aged seeds for 10 d and unaged seeds 2 d postgermination and roots analyzed by PI staining and confocal microscopy. Col-0 images include GFP signal from the *PWOX:GFP* QC reporter. Bar is 50 μm. (*E*) PCD was quantified as the percentage of roots displaying one or more dead cells in unaged seeds (black bars) and after 10 d aging (yellow bars). Significance groups are indicated by letters (*P* < 0.05, *n* > 40 per treatment, Fisher’s exact test with post hoc analysis).

Genome damage occurs naturally in response to seed aging, suggesting that the plant DDR is important to seed longevity (27). In seedlings, the transcription factor SOG1 integrates ATM and ATR signaling, including activation of PCD, the transcriptional DDR, and cell cycle regulation (4–6). We investigated the germination performance of wild-type and *sog1* mutant seed using accelerated aging (35 °C and 83% relative humidity), widely used to simulate the natural aging process (28). Germination of unaged wild-type (Col-0) *Arabidopsis* seeds and the *sog1* mutant seed displayed no significant difference (Fig. 3*A*). However, after 7 d of accelerated aging, *sog1* mutant seed exhibited higher levels of germination than wild-type controls (Fig. 3*B*) (*P* < 0.05). While Col-0 seeds display 10% viability after 14 d of aging, *sog1-2* and *sog1-3* lines maintain 40% viability (*P* < 0.01), phenocopying *atm* and *atr* (8). However, seedlings germinated on filter paper from 14-d-old *sog1* seed displayed reduced survival after transfer to soil in comparison to their wild-type counterparts (*P* < 0.01), demonstrating the consequences of SOG1 cell cycle checkpoint deficiency on subsequent seedling growth (Fig. 3*C*). We next investigated the incidence of SOG1-mediated PCD in germinating wild-type and *sog1* mutant seeds using PI viability staining of *Arabidopsis* embryos 2 d postgermination, when PCD was observed in irradiated seeds. The activity of PCD in response to genome stress in seeds is poorly defined, although these pathways have the potential to significantly affect seed and seedling vigor. Furthermore, the role of PCD in seed aging remains unknown. No significant incidence of cell death was observed in high-quality, unaged wild-type or *sog1* mutant seed (Fig. 3 *D* and *E*). However, in seed lots aged for 10 d (60% final viability), PCD could be observed in 30% germinating wild-type seeds. In aged *sog1* mutant seed, there was a significant reduction in roots displaying cell death (*P* < 0.01, Fisher’s exact test), with 5% roots displaying dead cells in the RAM of the embryo. In addition, aged *atm* and *atr* mutant seeds also displayed significantly reduced PCD relative to wild type, consistent with the function of SOG1 downstream of both ATM and ATR in seed longevity. In contrast, even unaged *lig4 lig6* seeds, deficient in DSB repair by both LIG4 and LIG6, displayed significantly enhanced levels of PCD relative to wild type, indicating that PCD is activated by DSBs incurred in seeds. Furthermore, naturally aged *lig4 lig6* lines (9 y postharvest, >95% viability) displayed significantly elevated levels of PCD relative to unaged and wild type (WT) seeds. Collectively, these results indicate that SOG1 activation results in PCD in aged seeds and delays germination, contributing to successful seedling establishment.

### DNA Repair Pathways Required for Seed Longevity.

The striking insensitivity to DNA damage displayed by seeds early in germination is consistent with the completion of DNA repair before cell cycle activation. This minimizes the mutational and growth-inhibitory effects of DNA damage accumulated in desiccation and quiescence (10). Here, we investigated the requirement of specific DNA repair pathways in seed longevity through genetic analysis of mutant lines deficient in core factors representing the major pathways. All mutant lines were analyzed together to enable comparative analysis of their contributions to germination performance. The sensitivity of mutant lines to accelerated aging (35 °C and 83% relative humidity (RH)) in comparison to wild type was investigated using the following mutant alleles: *ku70-1* and *ku80-3* (NHEJ); *xrcc2-1*, *-2*, and *-3* (homologous recombination [HR]); *arp1-1* and *arp-2* (BER); and *ercc1-1* and *ercc1-2* (nucleotide excision repair [NER]).

**Fig. 4.**
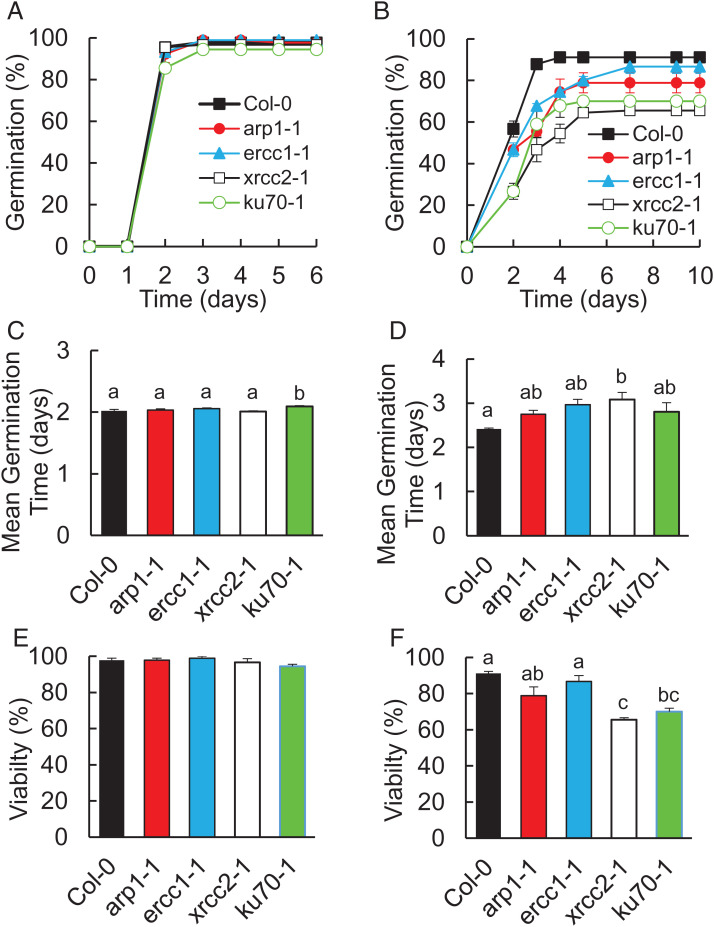
Germination performance of DNA repair mutant and wild-type lines. Aging sensitivity of independent alleles of mutants in the major plant DNA repair pathways relative to wild-type lines. Germination of Col-0 and mutant lines was analyzed after accelerated aging at 35 °C and 83% RH relative to unaged control seed. Seeds were stratified at 4 °C for 2 d before transfer to 23 °C 16-h day and scored for radicle emergence each day poststratification. (*A*) Germination of Col-0, *arp1-1*, *xrcc2-1, ercc1-1*, and *ku70-1* mutant alleles. (*B*) Germination of Col-0 and mutant alleles after aging for 1 wk . (*C*) MGT of control seed lots. (*D*) Mean germination time of 7-d aged seed lots. (*E*) Mean viability of control seed lots. (*F*) Mean viability of 7-d aged seed lots. Error bars represent the SEM of the mean of three replicates of 30 seeds. Data were analyzed by ANOVA with Tukey’s post hoc correction for multiple testing (*P* < 0.05).

Unaged high-quality wild-type and mutant lines all displayed similar germination profiles (Fig. 4 *A*, *C*, and *E* and *SI Appendix*, Figs. S7 and S8). However, accelerated aging for 7 d resulted in a slight delay to germination in mutant lines relative to wild-type controls, which was only significant in the HR-deficient *xrcc2* mutant lines (*P* < 0.05, ANOVA). Lines deficient for DSB repair by either HR or NHEJ pathways displayed significantly greater loss of viability relative to wild-type controls (*P* < 0.05, ANOVA) exhibiting c.25% lower viability than wild type (Fig. 4*F* and *SI Appendix*, Figs. S7 and S8). Deficiency in BER or NER, which repair single-stranded DNA damage, had less effect on germination potential, with only the *arp1-2* allele displaying significant hypersensitivity to seed aging. We conclude that all DNA repair pathways contribute to maintenance of seed longevity but that repair of cytotoxic DSBs is the most important for seed vigor and viability.

## Discussion

The desiccation-tolerant (orthodox) seed incurs remarkable levels of genome damage in desiccation and quiescence, which results in seeds aging, impacting seed quality (21). Seed longevity is considerably reduced under suboptimal environmental conditions, and understanding the molecular factors which determine seed quality is important for sustainable crop yields under changing climates. Here, we reveal that imbibed *Arabidopsis* seeds exhibit striking resistance to the effects of DNA damage which is lost as the embryo progresses to germination, reflected by key developmental and transcriptional differences in the DDR at the seed stage of the plant lifecycle. We demonstrate that SOG1 is a determinant of seedling vigor, and SOG1-mediated PCD occurs in response to seed aging. Taken together, our results suggest that low cell cycle activity and DDRs provide mechanisms that sustain growth and genome stability, mitigating the genome damage incurred during quiescence in this crucial phase of the plant’s lifecycle.

Here, we show that imbibed *Arabidopsis* seeds display little incidence of PCD and meristem disruption in response to DNA damage. This contrasts markedly to the DDRs previously observed in seedling development where genotoxic stress leads to PCD and meristem arrest and reorganization (7). In seedlings, expression of the QC marker WOX5 expands into neighboring cells, and the RAM becomes disorganized, accompanied by extensive cell death. Within 7 d of recovery in the absence of the genotoxin, wild-type roots subsequently reestablish meristem function allowing normal growth to resume, with similar activities observed in lateral root meristems (29). The plastic nature of plant root system architecture facilitates permanent arrest of root growth that intercepts soil contaminated with genotoxins (e.g., heavy metals), thereby promoting root development into more favorable soils. This contrasts with the germinating seed, which is highly dependent on the growing radicle for the essential water and nutrients that support early growth and seedling establishment. Additionally, the mechanical strength of the embryonic radicle to push through the testa and endosperm is an important factor that determines both germination vigor and also postgermination for the root to mechanically push through soil in seedling establishment (10, 30). Thus, the loss and replacement of compromised cells through PCD has the potential in the short term to negatively influence the mechanical properties of the root to drive germination and promote successful seedling establishment. Our findings establish that SOG1-mediated PCD occurs in response to naturally occurring DNA damage in aging seeds, revealing the physiological significance of the DDR in early plant development. However, we show that PCD in *Arabidopsis* seeds is largely postgerminative and of lower incidence relative to seedling roots. These results are in line with previous reports that have identified evidence of PCD in aged seeds, including DNA laddering in aged pea seeds and DNA breaks identified by terminal deoxynucleotidyl transferase dUTP nick-end labeling in sunflower seeds (31, 32).

*Sog1* mutant seeds deficient in DNA damage cell cycle checkpoint activity display apparently increased seed viability levels relative to wild type after aging. However, the resulting seedlings exhibit reduced survival on soil in comparison to their wild-type counterparts, consistent with the failure to impose a damage-induced lag phase to germination (16). This indicates that SOG-mediated DDRs are important in germination, in line with previous observations demonstrating important roles for ATM in seeds (8). Failure to activate DNA damage-induced cell cycle checkpoints in plants leads to the accumulation of increased levels of growth-inhibitory lesions and heritable mutations, with the potential to alter species fitness in the longer term (1). Thus, SOG1 and the DDR function to minimize the consequences of genome damage accumulated in quiescence on future plant growth. Seed aging is associated with an enhanced requirement for DSB repair and DDR factors in order to prolong seed viability and promote seedling establishment.

The lag period between imbibition and cell cycle activation as desiccation-tolerant seeds progress to germination provides an important window of opportunity for DNA repair, helping to minimize the potential genetic damage that results from DNA replication or mitosis in the presence of DNA lesions (16). Here, analysis of DNA repair-deficient *Arabidopsis* seeds identified that the major DNA repair pathways are important for maintenance of seed longevity but that DSB repair by both HR and NHEJ were the major determinants of recovery from seed aging. This supports our previous report demonstrating a requirement for the DSB factors DNA ligase 4 and 6 in seed longevity (9). The requirement for HR is consistent with the presence of DNA lesions that interfere with S-phase. DNA replication initiates prior to germination, and cell cycle progression is an important driver of germination (15). Seeds were previously demonstrated to require HR, as germination of X-ray–treated RAD51-deficient maize seed was delayed by 3–4 d, and germinated seedlings did not survive past 2 wk, in contrast to wild-type lines, which survived to develop into healthy seedlings (33). These results highlight physiological roles for the DDR and the developmental significance of plant genome maintenance factors, mutants of which are typically aphenotypic under standard growth conditions.

Natural plant populations need to adapt to rapidly changing environmental conditions associated with climate change in order to maintain ecosystem fitness, and germination is particularly susceptible to abiotic stresses, including warming and drought (34). These studies have significant implications for long-term seed storage and genetic stability of plant populations under changing climates (35). Furthermore, understanding the DNA repair processes important to seed germination performance and viability has potential applications in the development of predictive markers for seed lot quality and genetic improvement of crop resilience to seed stresses.

## Materials and Methods

### Plant Material and Growth Conditions.

*Arabidopsis* plants were raised in growth chambers under constant humidity (30%), with 16-h light and 8-h dark cycles at 23 °C. *Arabidopsis* Col-0 and mutants were obtained from Nottingham *Arabidopsis* Stock Centre (NASC). Plants were grown on half-strength MS, 1% sucrose, 0.5 g l^−1^ 2-(N-morpholino)ethanesulfonic acid (MES) and 0.8% plant agar (Duchefa) pH 5.7 on 16 h:8 h light–dark cycles at 22 °C. X-ray treatment (100 Gy) was performed at ambient temperature in the dark using a dose rate of ∼2 Gy/min using a 160 kV RS-2000 X-Ray Irradiator (Rad Source). The following genotypes were obtained from the NASC: Col-0, *arp-1* (AT2G41460, SALK_021478), *ercc1-1* (AT3G05210, SALK_033397), *ku70-1* (AT1G16970, SALK_123114), *ku80-3* (AT1G48050, SAIL_714A04), and *xrcc2-1* (AT5G64520, SALK_029106) and are characterized in *SI Appendix*, Figs. S9–S12. For each experimental replicate, seeds from all lines were harvested simultaneously and stored at 15 °C and 15% humidity for 2 mo to allow after-ripening. Germination tests, accelerated aging, and viability staining were performed according to published protocols (9, 36, 37). Accelerated aging was performed at 35 °C and 83% relative humidity by incubating seeds over saturated KCl in a sealed container in an incubator. Mean germination time was calculated as described previously (38). *Arabidopsis* lines were as previously described *ercc1-1* (39), *arp-1* (40), *xrcc2-1* (41), *ku70-1* (42), *lig4-5, lig6-1*, and *lig6-1 lig4-5* (9). Generation of *sog1* and *xrcc2* CRISPR-Cas9 alleles were generated as described by Fauser et al. (23) and in *SI Appendix*, Fig. S2, and *sog1-2* was crossed into a *PWOX5:GFP* reporter (43).

### Plant Genotyping and Microscopy.

*Arabidopsis* DNA extraction for PCR genotyping was performed by grinding plant tissue in shorty buffer (0.2 m Tris pH 9.0, 1% sodium dodecyl sulfate (SDS), 0.4 m LiCl, 25 mm ethylenediaminetetraacetic acid (EDTA)) in a 1.5 mL microfuge tube using a plastic micropestle. Cell debris was pelleted at 13,000 g for 5 min and the supernatant mixed 1:1 with 100% isopropanol and DNA recovered by centrifugation and dissolved in TE buffer. Plant genotyping was performed by PCR (GoTaq, Promega) using primers designed by iSect software (signal.salk.edu/tdnaprimers.2.html) and insertion sites confirmed by sequence analysis (Genewiz). Confocal microscopy was performed using a Zeiss LSM700 inverted microscope.

### RNA Sequencing Analysis.

Samples were incubated at 30 min at RT either with or without exposure to 100-Gy X-rays, and RNA was isolated after a 6-h recovery at 23 °C in the light. Total RNA was isolated from ground tissue of whole 0-d seeds stratified at 4 °C for 2 d on half MS agar or 7-d seedlings grown on half MS agar using an SV RNA isolation kit (Promega). Library preparation and paired-end sequencing was conducted by Novogene. The qualities of the FASTQ files were assessed using FastQC (https://www.bioinformatics.babraham.ac.uk/projects/fastqc/). The further quality control procedure involves two steps. First, the adapter sequences among the reads were removed using Cutadapt (44). Second, the poor-quality sequences were trimmed and filtered according to quality scores using PRINSEQ (45). The clean reads were mapped onto the *Arabidopsis* reference genome (TAIR10) downloaded from Ensembl Plants (release 50) (46) using STAR (47), followed by converting, sorting, and indexing of the alignment files using SAMtools (48). Uniquely mapped reads were selected for any further analysis. The read counts for genes in the GTF file were generated with featureCounts() function of Rsubread package (49). Differentially expressed genes for each comparison were identified through the DESeq2 package (50). Venn diagrams were calculated using Biovenn (51), and gene ontology enrichment was performed using Araport (52). Confirmation was performed using qPCR with a CFX96 thermocycler and Ssofast SYBR green (BioRad). Data were normalized to *ACTIN7* and the ratio presented relative to the irradiated 7-d Col-0 seedlings (C) or control 7-d Col-0 seedlings (D). Primer sequences are presented in *SI Appendix*, Table S5.

### Statistical Analyses.

Data were analyzed using R (53). Kolmogorov-Smirnov (KS) tests (*P* > 0.05) were used to test for normality, homogeneous variance was determined using Levene’s test (*P* > 0.05), and log transformation was used as indicated. Student’s *t* test was used for pairwise comparisons. ANOVA with post hoc Tukey’s tests was used for multiple comparisons. Two-way ANOVA was used to test the interaction of two variables on a given parameter. A Mann–Whitney *U* test was used for analysis for data that were not normally distributed. Boxplots displayed the first to third quartiles, with whiskers showing the data range within 1.5-fold of the box height, and any data points outside this range were plotted as outliers.

## Supplementary Material

Supplementary File

Supplementary File

Supplementary File

## Data Availability

RNAseq data are available and have been deposited in the National Center for Biotechnology Information BioProject under accession no. PRJNA801609 (54).
