# Supplementary material for: Seed DNA damage responses promote germination and growth in Arabidopsis thaliana
Source: Proc Natl Acad Sci U S A. 2022 Jul 18;119(30):e2202172119. doi: 10.1073/pnas.2202172119 (PMC9335332; doi:10.1073/pnas.2202172119)
Supplement: Supplementary File [file pnas.2202172119.sapp.pdf]

Supplementary Information for
Seed DNA damage responses promote germination and growth in *Arabidopsis thaliana*

Waterworth WM, Latham R, Wang D, Alsharif M and West CE

West CE
Email:c.e.west@leeds.ac.uk

This PDF file includes:

Figures S1 to S12
Tables S1 to S5
SI References

Other supplementary materials for this manuscript include the following:

Datasets S1 to S2

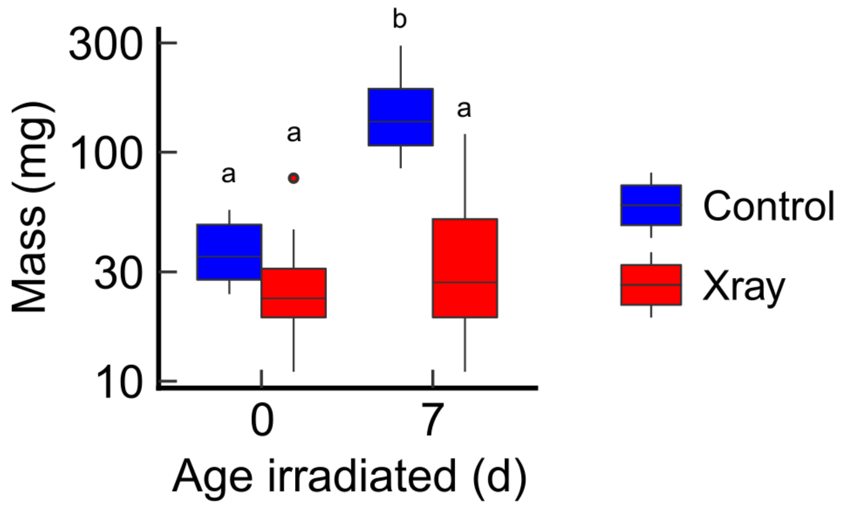

Fig. S1. Long term effects of X-irradiation on plant growth. Mass of above ground Arabidopsis fresh tissue 3-weeks post-irradiation (100Gy) of seeds at 0d (after 2d stratification at 4°C) or 7d seedlings, with unirradiated controls. Plants were grown on half-MS at 23°C 16h day. Letters indicate homogenous subsets (ANOVA on log transformed data with Tukey post-hoc testing, $p<0.001$, $n=17-21$)

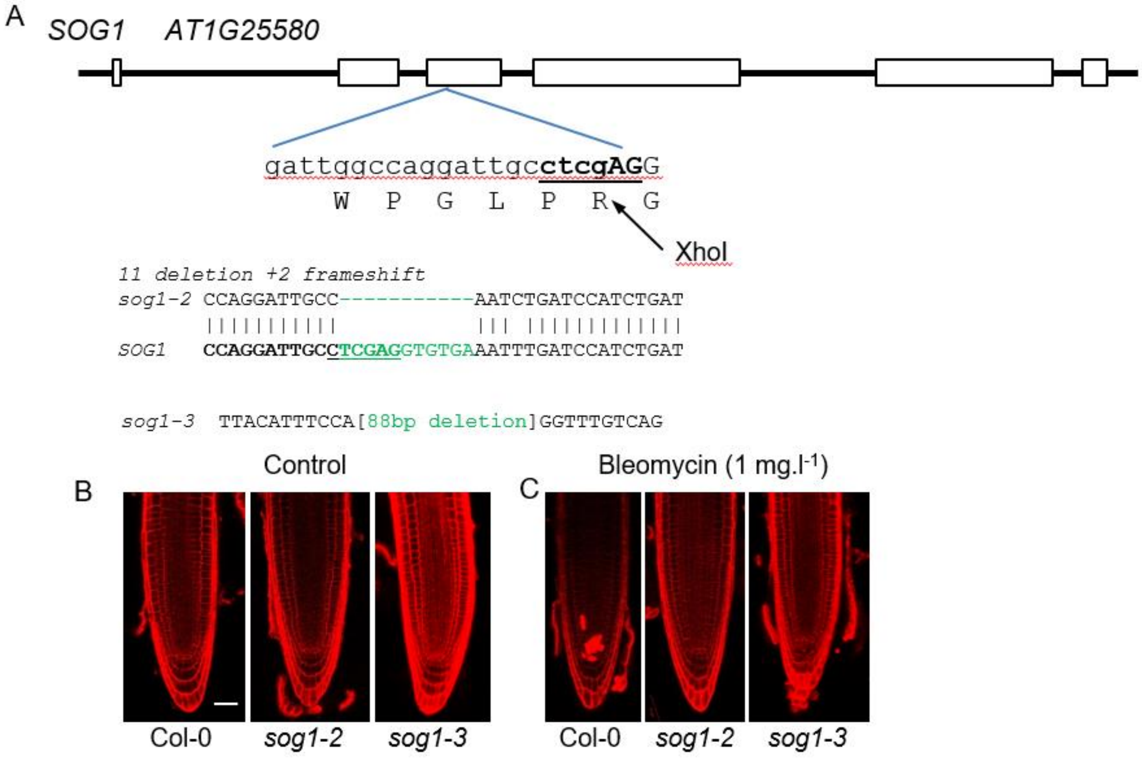

D

SogF TGCATTTGGGTCTTTCACTG
SogR GACACCGTCCCATCACTCTT
SOG Cas f ATTG GATTGGCCAGGATTGCCTCG
SOG Cas r AAAC CGAGGCAATCCTGGCCAATC

Fig. S2. Isolation of *sog1* mutant alleles. (**A**) Schematic of the *SOG1* gene showing the region targeted by the Cas9-sgRNA construct. Exons are shown as boxes. The protospacer and corresponding amino acid sequence is shown. The protospacer adjacent motif is shown in caps and the XhoI restriction site underlined. Transformed lines were selected on BASTA and screened by amplification of the *SOG1* locus with SogF/SogR primers followed by XhoI digestion. Lines that had lost the XhoI site were outcrossed to remove the Cas9 construct and homozygous mutant lines isolated. Sequence of *sog1-2* mutants revealed a 11bp deletion and a frameshift. Analysis of *sog1-3* mutants showed an 88bp deletion. **(B-C**) Phenotypic analysis of 7d wild type and *sog1* mutant lines to analyse the incidence of cell death. Arabidopsis plants were incubated on half-MS agar (**B**) or media supplemented with bleomycin (1mg.l^{-1}) to induce DNA damage (**C**) for 24h and roots analysed by staining with propidium iodide (10µg.ml^{-1}) and fluorescence microscopy (Zeiss LSM700) as described in the main methods. Cell death in wild type lines results in staining of stem cell initials, whereas this is absent in *sog1* mutant lines. Bar is 50µm (**D**) Primers used in the generation of the mutant lines using CRISPR/Cas9 as according to published protocols (1). SOG Cas f / r were used for generating the protospacer in pEnChimera. SogF/R were used for PCR screening Arabidopsis transformants for mutation in *SOG1* using loss of the XhoI site to identify mutants.

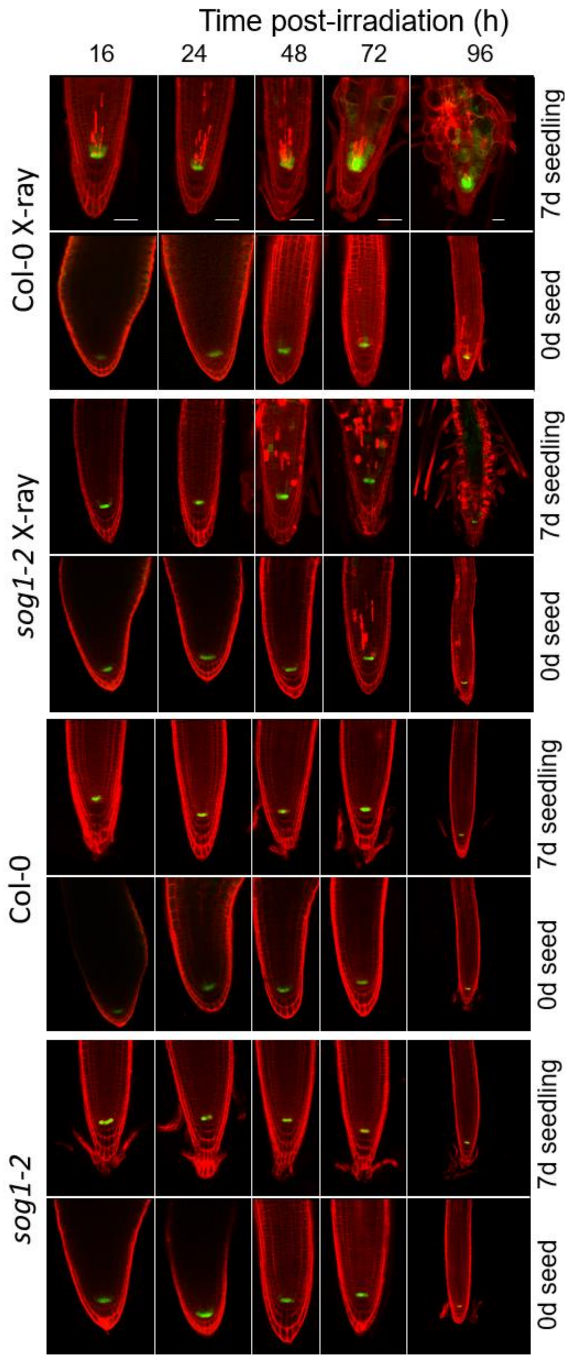

Fig. S3. X-ray induced programmed cell death in Arabidopsis seeds and seedlings. Representative confocal images of PI stained Arabidopsis Col-0 or *sog1-2* roots expressing the QC marker PWOX5:GFP. Seeds were stratified for 2d at 4°C and either exposed to 100Gy X-rays immediately post-stratification (0d seeds) or after 7d growth on half MS (7d seedlings). The incidence of cell death in roots was monitored over 96h recovery from irradiation. Scale bar: 50 µm. All images are at the same magnification apart from the 96h time point which is 0.5x the other time points. Quantification of multiple repeats is presented in Fig S4.

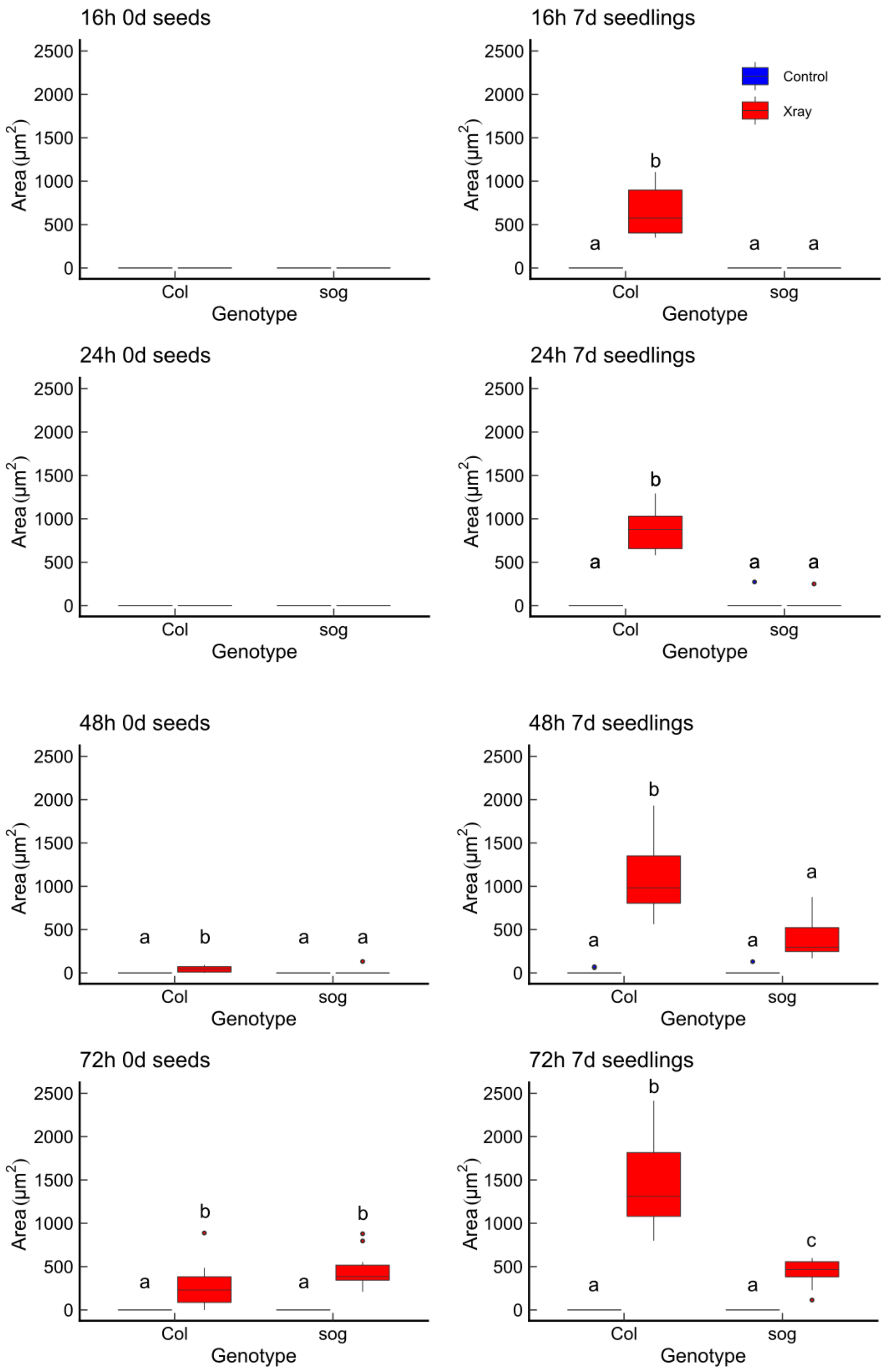

Fig. S4. Quantification of X-ray induced PCD in Arabidopsis roots. Cell death was induced as described for Fig S3. Arabidopsis Col-0 and *sog1-2* mutant seeds were stratified for 2d at 4°C before transfer to 23°C 16h day and either exposed to 100Gy X-rays immediately post-stratification (0d seeds) or after 7d growth on half MS and cell death (7d seedlings) and visualized by PI staining at the time indicated. The appearance of cell death was quantified through the z section with maximal PWOX5-GFP expression at the time indicated. Letters indicate homogenous subsets (ANOVA with Tukey post-hoc testing, $p<0.001$, $n=10$)

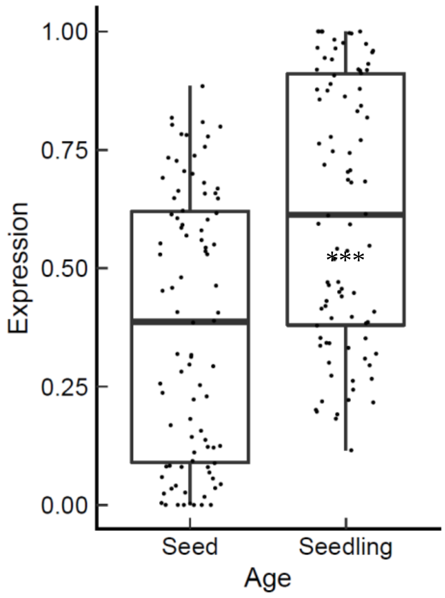

Fig. S5. Relative cell cycle gene expression in Arabidopsis Col-0 0d seeds and 7d seedlings. Seeds were stratified on half MS agar at 4°C for 2d and whole seeds or whole 7d seedlings used for RNA isolation and RNAseq analysis. Gene expression levels are expressed as the fraction of expression at each developmental stage and 90 core cell cycle factors are analyzed, including the following families: *CYCLINS, CYCLIN DEPENDENT KINASES, SIAMESE RELATED, CASEIN KINASE I-LIKE, KIP-RELATED PROTEIN and E2F, DP, RB, WEE1* and *CDC25.* ***$p<0.001$, Mann-Whitney U test, n=90.

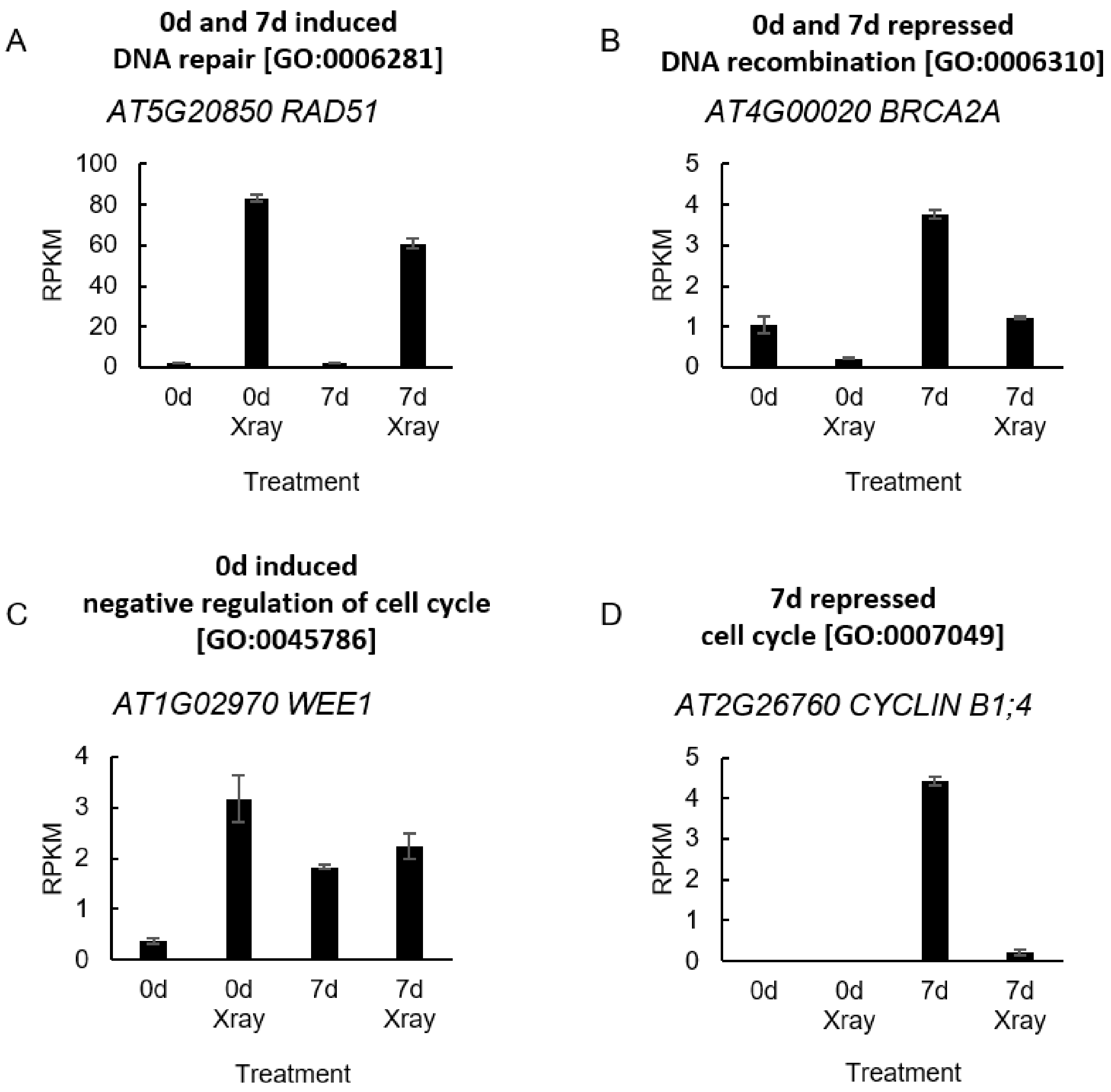

Fig. S6. Transcript counts (RNAseq) for genes representative of enriched gene ontology categories presented in Fig 2B. (**A**) Genes induced 6h post X-irradiation (100Gy) in both 0d stratified seeds and 7d seedlings include the DNA repair factor *RAD51*. (**B**) Genes with reduced levels after X-ray treatment at both developmental stages include DNA replication and recombination factors, with *BRCA2* involved in homologous recombination. (**C**) Negative regulators of the plant cell cycle including *WEE1* are specifically induced in 0d seeds in response to X-rays at the 6h time point. (**D)** 7d seedlings display a strong reduction of cell cycle gene transcript levels including *CYCLINB1;4* after irradiation, not observed in 0d seeds. Error bars show the range of values (n=2).

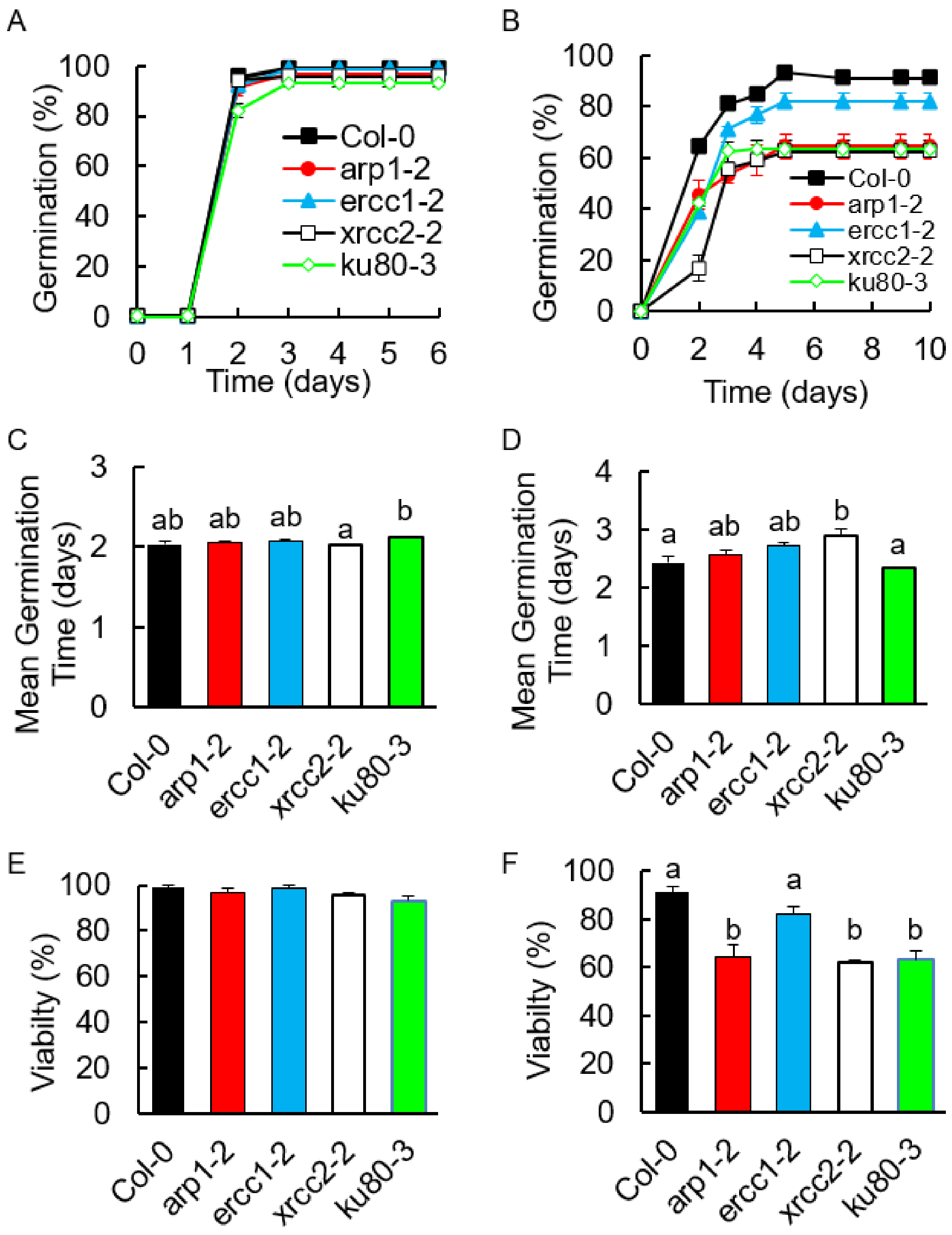

Fig. S7. Accelerated ageing sensitivity of independent alleles of mutants in the major plant DNA repair pathways. Germination of Col-0 and mutant lines was analyzed by accelerated ageing at 35°C and 83% RH relative to unaged control seed. Seeds were stratified at 4°C for 2d before transfer to 23°C 16h day and scored for radicle emergence each day post-stratification. (**A**) Germination of unaged Col-0, *arp1-2*, *xrcc2-2, ercc1-2* and *ku80* mutant alleles. (**B**) Germination of wild type and mutant alleles after ageing for 1 week at 35°C and 81% relative humidity. (**C**) Mean Germination Time of control (unaged) seeds lots. (**D**) Mean Germination Time after ageing treatment. (**E**) Mean viability of control seeds lots. (**F**) Mean viability of aged seeds lots. Data was analyzed by ANOVA with Tukey post hoc correction for multiple testing ($p<0.05$). Error bars are SEM. n=3 replicates of 30 seeds.

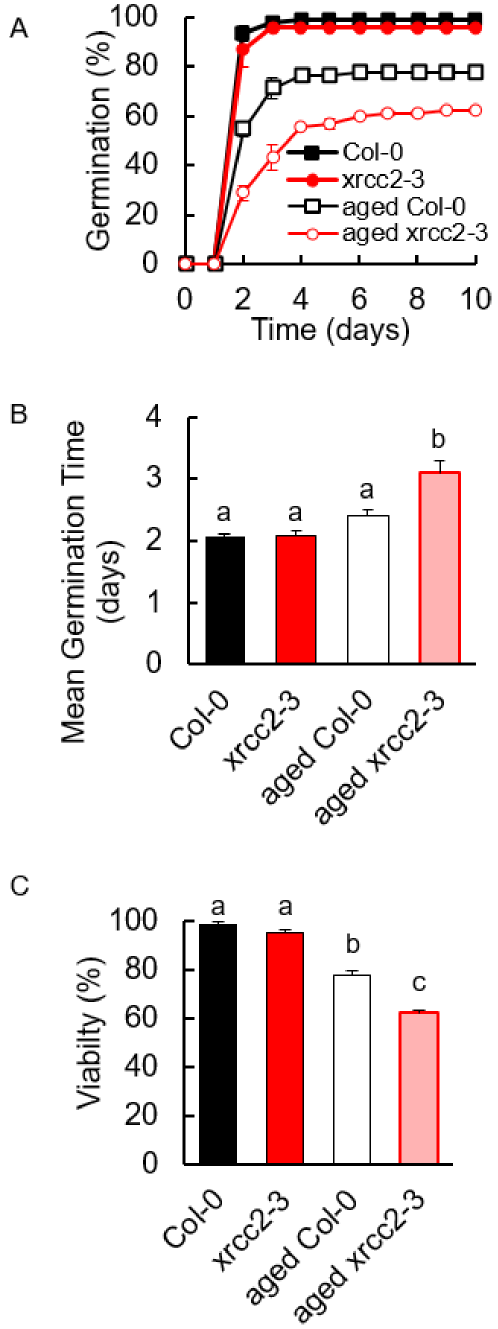

Fig. S8. Accelerated ageing sensitivity of Arabidopsis Col-0 and *xrcc2-3* mutants. Germination of Col-0 and mutant lines was analyzed by accelerated ageing for 7d at 35°C and 83% RH relative to unaged control seed. Seeds were stratified at 4°C for 2d before transfer to 23°C 16h day and scored for radicle emergence each day post-stratification. (**A**) Germination of Col-0 and a CRISPR/Cas9 generated *xrcc2-3* mutant line with and without accelerated ageing. (**B**) Mean Germination Time and (**C**) mean viability calculated from the data presented in (**A**) Data was analyzed by ANOVA with Tukey post hoc correction for multiple testing ($P<0.05$). Two-way ANOVA of data presented in (**B**) and **(C)** indicated a significant effect of genotype on the sensitivity to seed ageing ($p<0.001$). Error bars are SEM. n=3 replicates of 30 seeds.

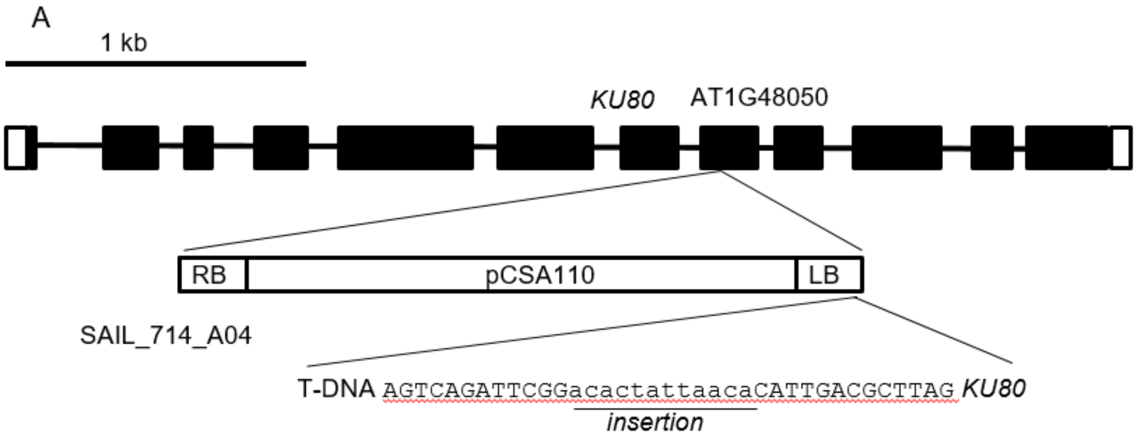

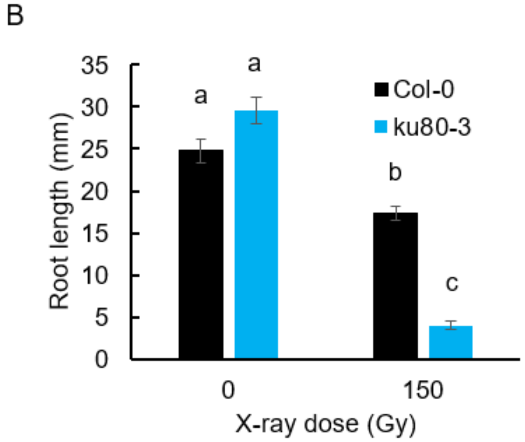

Fig. S9. Isolation of the *ku80-3* mutant allele. (**A**) Schematic of the *KU80* gene showing site of insertion in the T-DNA mutant line SAIL_714_A04. Exons are shown as boxes. A 12 bp insertion is located between the T-DNA and *KU80* (**B**) Sensitivity of the *ku80-3* mutant line to 100Gy X-irradiation, quantified by final root growth 10d post-irradiation after treatment of 2d stratified seeds. Data was analyzed by ANOVA with Tukey post hoc correction for multiple testing ($p<0.01$). Error bars are SEM, n=21-36.

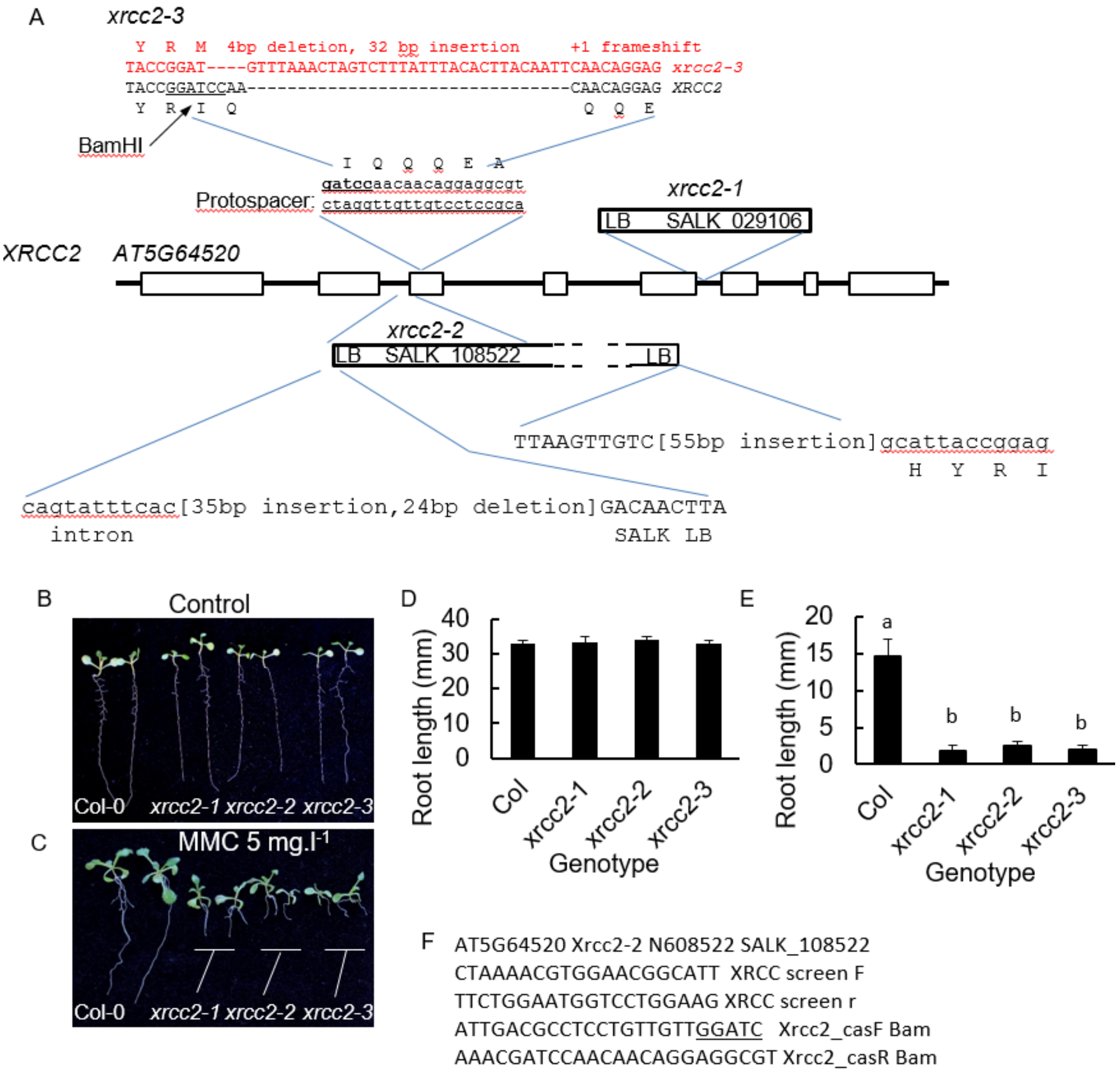

Fig. S10. Isolation of *xrcc2* mutant alleles. (**A**) Schematic of the *XRCC2* gene showing the positions of the *xrcc2-1*, *2-2* and *2-3* mutant alleles. Exons are shown as boxes. The *xrcc2-2* allele (SALK_108522) has a complex T-DNA insertion including sequence insertions of unknown origin on both the 3' and 5' insertion borders. The insertion results in a 24bp deletion across an intron-exon boundary. The *xrcc2-3* allele is generated using CRISPR-Cas9 mutagenesis (Fauser et al (2016)). The mutant allele contains a 32bp insertion and a 4bp deletion that removes a BamHI site and results in a frameshift. (**B-E**) Phenotypic analysis of wild type and *xrcc2* mutant lines. (**B**) growth under control conditions (**C**) growth in the continuous presence of mitomycin C (5 mg.l^{-1}). (**D-E**) Quantification of root growth in control conditions (**D**) and (**E**) in the continuous presence of 5 mg.l^{-1} mitomycin C (**F**) Primers used in the isolation of the mutant lines. Error bars are SEM, n=8-14.

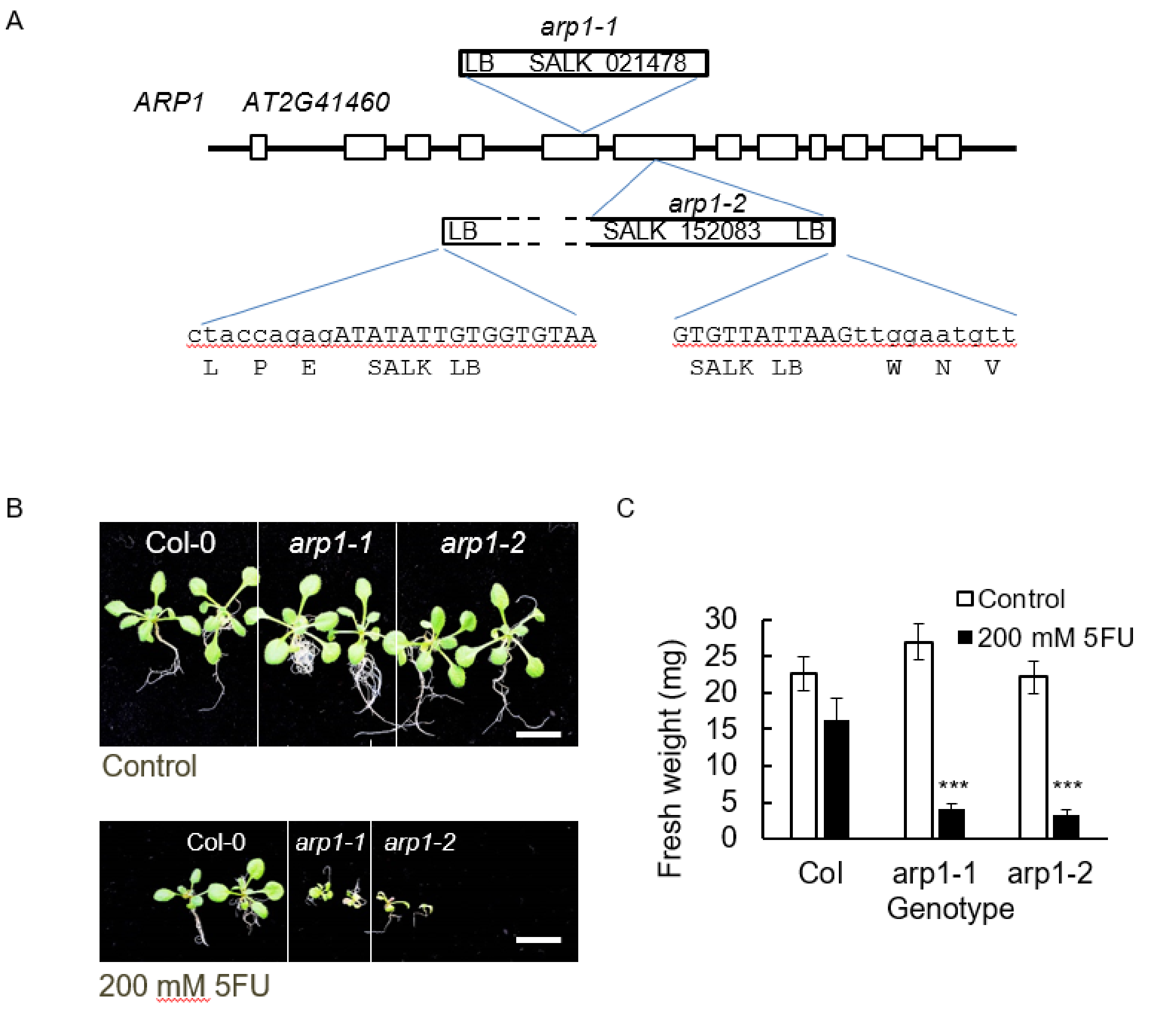

D

arp1-2 AT2G41460

NASC ID: N680401 Name: SALK_152083C

LP CGTTCGGATAGTGTCACCATC

RP CCCATTCTTCGATCCTGTATG

Fig. S11. Isolation of *arp1* mutant alleles. (**A**) Schematic of the *ARP1* gene showing the positions of the *arp1-1* and *1-2* mutant alleles. Exons are shown as boxes. The *arp1-2* allele (SALK_152083) is in exon 6. (**B**) Phenotypic analysis of wild type and *arp1* mutant lines. Seeds were stratified for 2d at 4°C in sdH_2O before transfer to agar plates. Control plants were grown on half MS media at 23°C 16h day and treated plants were grown in media supplemented with 200mM 5-fluorouracil (5-FU). Plant growth was monitored after 2 weeks (**C**) Quantification of growth (**D**) Primers used in the isolation of the mutant lines. ***p<0.001 T-Test. Error bars are SEM, n=5-8.

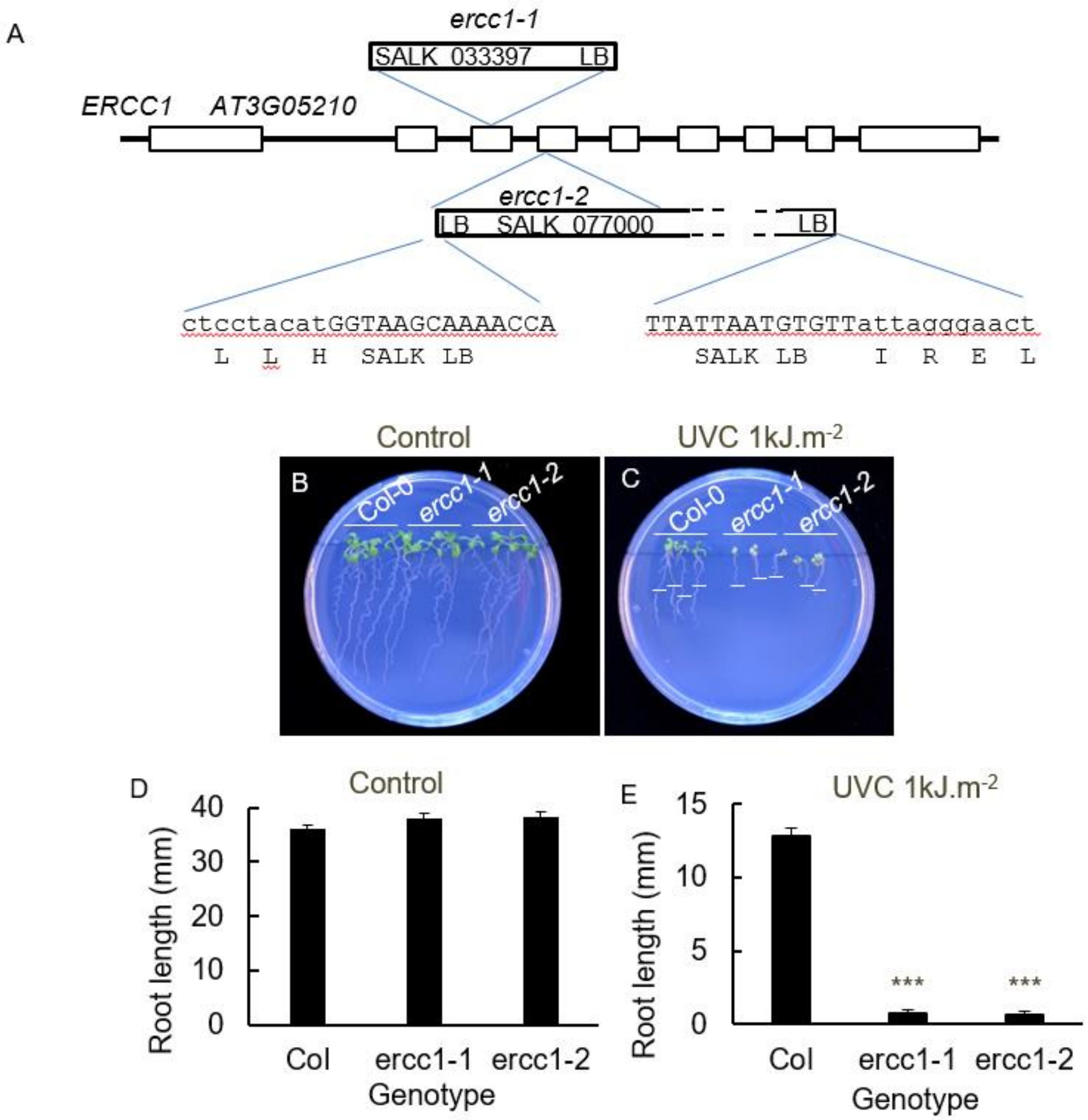

F

SALK T-DNA Homozygous Knockout Line for At3g05210
NASC ID: N663022 Name: SALK_077000C
GTGCCATCGTACCAGGAAGT ercc mut F
TCGAGTCCTCCACAAAATCC ercc mut r

Fig. S12. Isolation of *ercc1* mutant alleles. (**A**) Schematic of the *ERCC1* gene showing the positions of the *ercc1-1*, and *1-2* mutant alleles. Exons are shown as boxes. The *ercc1-2* allele (SALK_077000) is in exon 4. (**B-E**) Phenotypic analysis of wild type and *ercc1* mutant lines. Quantification of growth in control plants at 14d (**B**) and plants exposed to 1kJ.m^{-2} UVC at the points indicated by the horizontal line. Plants were irradiated after 7d and grown for a further 7d post-treatment (**C**). Root growth is quantified in (**D**) control (n=10) and (**E**) irradiated (n=13-16). (**F**) Primers used in the isolation of the *ercc1-2* mutant line. *** p<0.001, T-Test. Error bars are SEM.

Table S1. Gene ontology enrichment in transcripts increased in X-ray treated 0d seeds and 7d seedlings displayed in Fig 2B.

**Increased in Xray treated 0d seeds and 7d seedlings**		
GO category	p adj	n
DNA repair [GO:0006281]	7.91E-15	28
cellular response to DNA damage stimulus [GO:0006974]	8.78E-15	29
DNA metabolic process [GO:0006259]	3.83E-14	32
double-strand break repair [GO:0006302]	5.3E-09	15
cellular response to stress [GO:0033554]	9.46E-07	36
DNA recombination [GO:0006310]	1.09E-06	15
response to ionizing radiation [GO:0010212]	2.95E-05	7
DNA replication [GO:0006260]	4.76E-05	13
double-strand break repair via homologous recombination [GO:0000724]	5.98E-05	10
cell cycle [GO:0007049]	6.31E-05	23
recombinational repair [GO:0000725]	9.76E-05	10
nucleic acid metabolic process [GO:0090304]	0.000334	64
meiotic cell cycle [GO:0051321]	0.000827	12
cell cycle process [GO:0022402]	0.000993	18
nucleobase-containing compound metabolic process [GO:0006139]	0.004644	65
meiotic nuclear division [GO:0140013]	0.005225	9
organelle fission [GO:0048285]	0.009457	12
nuclear division [GO:0000280]	0.009768	11
meiotic cell cycle process [GO:1903046]	0.010096	10
meiosis I [GO:0007127]	0.02081	7
heterocycle metabolic process [GO:0046483]	0.025043	66
DNA-dependent DNA replication [GO:0006261]	0.029887	9
meiosis I cell cycle process [GO:0061982]	0.03213	7
cellular aromatic compound metabolic process [GO:0006725]	0.040617	67

Table S2. Gene ontology enrichment in transcripts increased in X-ray treated 0d seeds displayed in Fig 2B.

**Increased in Xray treated 0d seeds**		
GO category	p adj	n
cell cycle [GO:0007049]	9.02E-07	26
meiotic cell cycle [GO:0051321]	1.49E-05	14
negative regulation of cell cycle [GO:0045786]	1.96E-05	10
negative regulation of cellular component organization [GO:0051129]	3.11E-05	10
regulation of cell cycle [GO:0051726]	3.29E-05	15
negative regulation of nuclear division [GO:0051784]	4.7E-05	7
cell cycle process [GO:0022402]	5.76E-05	20
negative regulation of organelle organization [GO:0010639]	0.000178	8
regulation of organelle organization [GO:0033043]	0.000475	13
regulation of nuclear division [GO:0051783]	0.001345	7
regulation of meiotic cell cycle [GO:0051445]	0.001689	5
nuclear division [GO:0000280]	0.001789	12
negative regulation of cell cycle process [GO:0010948]	0.002343	7
negative regulation of mitotic cell cycle [GO:0045930]	0.002343	7
DNA metabolic process [GO:0006259]	0.002738	19
negative regulation of cellular process [GO:0048523]	0.009391	22
cell cycle checkpoint [GO:0000075]	0.011763	6
organelle fission [GO:0048285]	0.013184	12
negative regulation of mitotic nuclear division [GO:0045839]	0.016819	5
regulation of cellular component organization [GO:0051128]	0.023126	15
cellular response to DNA damage stimulus [GO:0006974]	0.023809	15
cellular response to stress [GO:0033554]	0.044857	28

Table S3. Gene ontology enrichment in transcripts decreased in X-ray treated 0d seeds and 7d seedlings displayed in Fig 2B.

**Decreased in Xray treated 0d seeds and 7d seedlings**		
GO category	p adj	n
DNA replication [GO:0006260]	1.81E-09	8
DNA recombination [GO:0006310]	2.72E-05	6
cell cycle [GO:0007049]	4.3E-05	8
double-strand break repair via homologous recombination [GO:0000724]	4.37E-05	5
recombinational repair [GO:0000725]	5.63E-05	5
double-strand break repair [GO:0006302]	0.000268	5
DNA-dependent DNA replication [GO:0006261]	0.000366	5
DNA metabolic process [GO:0006259]	0.000397	7
DNA repair [GO:0006281]	0.001166	6
cellular response to DNA damage stimulus [GO:0006974]	0.002021	6

Table S4. Gene ontology enrichment in transcripts decreased in X-ray treated 0d seeds and 7d seedlings displayed in Fig 2B.

**Decreased in Xray treated 7d seedlings**		
GO category	p adj	n
cell cycle [GO:0007049]	1.16E-39	74
cell cycle process [GO:0022402]	3.45E-38	64
cell division [GO:0051301]	9.67E-31	55
mitotic cell cycle process [GO:1903047]	2.17E-30	43
mitotic cell cycle [GO:0000278]	2.84E-30	46
microtubule-based process [GO:0007017]	1.05E-20	35
regulation of cell cycle [GO:0051726]	4.31E-20	35
mitotic cell cycle phase transition [GO:0044772]	4.69E-19	23
microtubule-based movement [GO:0007018]	1.12E-18	21
cell cycle phase transition [GO:0044770]	2E-18	23
movement of cell or subcellular component [GO:0006928]	8.36E-16	21
regulation of transferase activity [GO:0051338]	5.93E-13	21
regulation of protein kinase activity [GO:0045859]	1.52E-11	18
regulation of kinase activity [GO:0043549]	2.26E-11	18
regulation of cell cycle process [GO:0010564]	4.34E-11	20
regulation of cyclin-dependent protein serine/threonine kinase activity [GO:0000079]	4.39E-11	15
regulation of cyclin-dependent protein kinase activity [GO:1904029]	4.39E-11	15
nuclear division [GO:0000280]	5.73E-11	24
regulation of protein phosphorylation [GO:0001932]	1.42E-10	18
cytokinesis [GO:0000910]	2.01E-10	18
regulation of phosphorylation [GO:0042325]	7.37E-10	18
microtubule cytoskeleton organization [GO:0000226]	1.43E-09	21
regulation of protein serine/threonine kinase activity [GO:0071900]	1.64E-09	15
organelle fission [GO:0048285]	3.82E-09	24
cytokinesis by cell plate formation [GO:0000911]	1.02E-08	14
regulation of protein modification process [GO:0031399]	3.87E-08	21
regulation of mitotic cell cycle [GO:0007346]	3.91E-08	15
meiotic nuclear division [GO:0140013]	2.17E-07	16
regulation of phosphate metabolic process [GO:0019220]	2.31E-07	18
regulation of phosphorus metabolic process [GO:0051174]	2.56E-07	18
meiotic cell cycle process [GO:1903046]	4.73E-07	18
meiotic cell cycle [GO:0051321]	1.15E-06	19
regulation of mitotic cell cycle phase transition [GO:1901990]	1.28E-06	11
regulation of cell cycle phase transition [GO:1901987]	2.48E-06	11
spindle organization [GO:0007051]	1.41E-05	11
male meiotic nuclear division [GO:0007140]	3.53E-05	8
triterpenoid metabolic process [GO:0006722]	4.32E-05	7
cytoskeleton organization [GO:0007010]	4.99E-05	21
microtubule cytoskeleton organization involved in mitosis [GO:1902850]	8.6E-05	8
regulation of catalytic activity [GO:0050790]	9.29E-05	22
chromosome segregation [GO:0007059]	0.000165	14
nuclear chromosome segregation [GO:0098813]	0.0002	13
regulation of molecular function [GO:0065009]	0.000241	22

tricyclic triterpenoid metabolic process [GO:0010683]	0.000308	4
cytoskeleton-dependent cytokinesis [GO:0061640]	0.000634	9
meiosis II [GO:0007135]	0.000686	7
meiosis II cell cycle process [GO:0061983]	0.000686	7
mitotic spindle organization [GO:0007052]	0.000903	7
regulation of G2/M transition of mitotic cell cycle [GO:0010389]	0.000903	7
chromosome separation [GO:0051304]	0.001113	8
regulation of cell cycle G2/M phase transition [GO:1902749]	0.001174	7
G2/M transition of mitotic cell cycle [GO:0000086]	0.001512	7
mitotic nuclear division [GO:0140014]	0.001607	11
sister chromatid segregation [GO:0000819]	0.001813	10
cell cycle G2/M phase transition [GO:0044839]	0.002434	7
chromosome organization [GO:0051276]	0.003757	27
male meiosis cytokinesis [GO:0007112]	0.004495	4
organelle organization [GO:0006996]	0.006025	55
regulation of cellular protein metabolic process [GO:0032268]	0.01002	22
mitotic cytokinesis [GO:0000281]	0.010156	7
cell cycle checkpoint [GO:0000075]	0.020205	7
histone phosphorylation [GO:0016572]	0.020465	4
thalianol metabolic process [GO:0080003]	0.020542	3
regulation of protein metabolic process [GO:0051246]	0.021588	22
spindle assembly [GO:0051225]	0.027712	7
meiotic cytokinesis [GO:0033206]	0.036375	4

Table S5. Primer sequences for qPCR

AT2G25060	GCTCAGTTAGGCTTGGTGGT
AT2G25060	AGCAAGGAGCCAAAGGACAA
AT5G06150	TGTACACTGCACGTAGCCTG
AT5G06150	CGTTCTGTCCCTCCATGCTT
AT4G05380_F	CCGAGAACCGCTCCATTCTT
AT4G05380_R	GCAATGCCGGATCGAGTTTC
AT2G18193_F	GATGGAATGGCTGGTGGGAA
AT2G18193_R	TTCCTGGTGGCCCATACAAC
ACTIN7_F	CCATCGCTCATCGGAATGGA
ACTIN7_R	TGGAACCACCACTGAGAACG
AT5G48720_F	TGATGCTGAGACTCCAATGC
AT5G48720_R	AGCTTTGAAACTGGCCTTGA

Dataset S1 (separate file).

Cell cycle gene expression (RPKM) in 0d seeds and 7d seedlings in the absence of additional treatments. 0d seeds were stratified for 2 days at 4°C on half-MS media and incubated at 23°C in the light for 6h. 7d seedlings were incubated for 7d at 23°C in 16h days. Relative expression of cell cycle genes between seeds and seedlings is calculated using the mean of two replicates. 90 core cell cycle factors are analyzed, including the following families: CYCLINS, CYCLIN DEPENDENT KINASES, SIAMESE RELATED, CASEIN KINASE I-LIKE, KIP-RELATED PROTEIN and E2F, DP, RB, WEE1 and CDC25.

Dataset S2 (separate file).

RPKM tab: Gene expression (RPKM) in 0d seeds and 7d seedlings 6h after irradiation with 100 Gy X-rays or unirradiated controls. 0d vs 7d tab: expression levels of transcripts that display significant changes in expression on irradiation at either 0d or 7d growth stages (Log2 fold change > 1 or < -1, adjusted p <0.05). Column headers are defined as:
0d: RPKM values in control seeds 6 hours post-stratification
7d: RPKM values in in control 7d seedlings
Xray_0d: RPKM values in stratified seeds 6h post-irradiation
Xray_7d: RPKM values in 7d seedlings 6h post-irradiation
0d vs X0d log2FoldChange = comparison of gene expression values in control seeds 6 hours post-stratification versus stratified seeds 6h post-irradiation
0d vs X0d padj = significance of differences in gene expression values in control seeds versus irradiated seeds. P value calculated DESeq2
7d vs X7d log2FoldChange = comparison of gene expression values in control seedlings versus 7d seedlings 6h post-irradiation
7d vs X7d padj= significance of differences in gene expression values in control seedlings versus irradiated seedlings. P value calculated DESeq2
X0d vs X7d log2FoldChange = comparison of gene expression values in stratified seeds 6h post-irradiation versus 7d seedlings 6h post-irradiation
X0d vs X7d padj= significance of differences in gene expression values in irradiated seeds versus irradiated seedlings. P value calculated DESeq2
Gene descriptions were obtained from Arabidopsis.org.

SI References

1. Fauser F, Schiml S, & Puchta H (2014) Both CRISPR/Cas-based nucleases and nickases can be used efficiently for genome engineering in Arabidopsis thaliana. *The Plant Journal* 79(2):348-359.
